# Too anxious to control: the relation between math anxiety and inhibitory control processes

**DOI:** 10.1038/s41598-020-76920-7

**Published:** 2020-11-16

**Authors:** E. Van den Bussche, K. Vanmeert, B. Aben, D. Sasanguie

**Affiliations:** 1 Brain & Cognition, KU Leuven, Leuven, Belgium; 2 Faculty of Psychology and Educational Sciences, Vrije Universiteit Brussel, Brussels, Belgium; 3 Department of Experimental Psychology, Ghent University, Ghent, Belgium; 4 Research Centre for Learning in Diversity, HOGENT, Ghent, Belgium

**Keywords:** Psychology, Human behaviour

## Abstract

Based on the attentional control theory, math anxiety has been explained in terms of impaired inhibition, a key cognitive control function associated with the central executive. Inhibition allows us to suppress task-irrelevant interference when needed. Inspired by the Dual Mechanisms of Control theory, the current study aimed to disentangle the effect of math anxiety on two cognitive control aspects that can be identified in inhibition. Reactive control occurs after interference is detected and is mostly used in a context where interference is scarce. Proactive control is used to prevent and anticipate interference before it occurs and is preferred in contexts where interference is frequent. We used an arrow flanker task where the proportion of interference was manipulated to stimulate the use of a reactive or proactive control strategy. The results showed that response times on trials containing interference increased with math anxiety, but only in a reactive task context. In a proactive task context response times were not influenced by math anxiety. Our results suggest that math anxiety impairs reactive control. We hypothesize that this finding can be explained by a higher state of distractibility, triggered both by the reactive context and by math anxiety.

## Introduction

The relation between anxiety and hampered cognitive performance has been addressed by several theoretical frameworks, one of the most influential being the attentional control theory (ACT; ^1^). The ACT, based on the processing efficiency theory ^2^, builds on the notion that the central executive is a crucial part of the working memory system ^3^. It is responsible for the storage and retrieval of information, but also for planning and decision-making. The ACT postulates that anxiety reduces precisely this central executive capacity. The theory states that anxiety biases attentional control, a key function of the central executive. As anxiety facilitates the detection and processing of danger or threat, it triggers attention allocation towards this threat. If these threat-related stimuli are irrelevant for the current task, this attention allocation will come at the expense of current task processing. Furthermore, high anxious people will consider it dangerous to allocate their full attention to only one task, but instead they will spread their attentional resources widely, which decreases their focus on the task at hand and makes them more vulnerable to distraction by irrelevant stimuli ^1^. According to the ACT, anxiety leads to performance decrements because of distraction from the task by irrelevant stimuli that can be external (e.g., distractors in the task) or internal (e.g., intrusive thoughts and worries).

Importantly, the ACT hypothesizes that anxiety impairs several (but not all) key executive functions associated with the central executive, a flexible supervisory system that controls and regulates cognitive processes ^3,4^. Specifically, it assumes that anxiety impairs executive functions that rely on attentional control. Based on the cognitive control functions of the central executive identified by Miyake and colleagues ^5^, the ACT proposes that inhibition in particular might be hampered by anxiety. The inhibition function of the central executive has been referred to as “negative” attentional control ^4^. Inhibition is a cognitive control process that allows us to suppress dominant, automatic responses when needed. Inhibition requires control in order to fence off interference by task-irrelevant information and to remain focused on the task at hand. Thus, the ACT assumes that anxiety will hamper performance on a task recruiting inhibition.

In the current study we focus specially on one form of anxiety, namely math anxiety. Math anxiety, a feeling of tension and apprehension surrounding the manipulation of numbers and solving of mathematical problems ^6^, is common and can have an enormous impact on people’s lives (e.g., ^7^). People who experience math anxiety tend to avoid situations where math skills are needed. This in turn has a negative influence on their math ability (i.e., the debilitating anxiety model, see for example ^8–12^), and can even lead them to preclude certain career choices ^13^. Although math anxiety overlaps with other anxiety constructs, such as test and general anxiety, it has been argued that it is a separate entity ^12,14–16^; see also a review by Ashcraft & Ridley ^17^). Math anxiety has been repeatedly linked to cognitive deficiencies, such as reduced working memory capacity ^9,18,19^ and attentional bias towards math-related information ^20,21^.

The ACT is also highly relevant for math anxiety and has received empirical support from this domain. For example, Hopko and colleagues ^22^ used a Stroop-like card task where adult participants had to inhibit the irrelevant content of the stimuli. The results showed that response time on this task was significantly worse for high than for low math anxious individuals. As this was the case for both letters and number stimuli, the authors suggested that a domain-general problem with inhibition might be at play in high math anxious individuals. Other adult studies reached the same conclusion ^19^. Furthermore, a negative relation has been reported between math anxiety and self-reported general inhibitory abilities ^23^. One possible explanation for the fact that math anxiety is related to impaired inhibition even in a math-unrelated context, might be that people who suffer from math anxiety, not only feel tensed when they are confronted with math content, but also when they need to perform a task which requires similar/overlapping cognitive processes as those involved in math (e.g. executive functions, such as inhibition; ^24,25^). Hence, any task (be it math-related or -unrelated) requiring similar/overlapping cognitive abilities that are also required to perform math, such as inhibition, might trigger math anxiety.

Recently, also in children, it was demonstrated that severe math anxiety is related to impaired general inhibition. Mammarella and colleagues ^26^ used two inhibition tasks. First, a “proactive interference task” was used to measure the resistance to *proactive* interference which was defined as the ability to delete information that has become irrelevant. Children remembered and recalled lists of words one by one. high math anxious children had more difficulty than children from the control group with resisting (now irrelevant) information from the previous list when recalling the current list. Second, the Hayling Sentence Completion task was also administered to measure the efficacy of prepotent response inhibition. Sentences were presented where the last word was missing and had to be completed either with a freely chosen expected word or with a freely chosen word that made the sentence meaningless. In the latter condition, children had to inhibit the dominant expected words, which were now irrelevant. This Hayling Sentence Completion task in fact measured a more *reactive* control component, which blocks dominant and prepotent cognitive responses that are automatically activated by the stimulus that is presented. The results showed that high math anxious individuals performed similarly to the control group children on this task and thus showed no difficulties with reactively inhibiting prepotent responses.

The study by Mammarella et al. suggests that not all aspects of inhibition might be affected equally by math anxiety. Although indirectly, we could infer from this study that proactive types of inhibition, where irrelevant information must be suppressed in advance of the stimulus, is more susceptible to the influence of math anxiety than reactive types of inhibition, where irrelevant information has to suppressed in reaction to the stimulus. Indeed, a more fine-grained link between math anxiety and inhibition control, by dividing this broad cognitive control process in a reactive and a proactive component, seems also theoretically plausible. According to the dual mechanism of control theory ^27,28^, proactive control will kick in preventively and anticipatory before interference occurs which places strong demands on working memory capacity. It is the preferred control strategy when interference is frequent and can be anticipated. Reactive control, on the other hand, detects and resolves interference only after its onset and is less resource consuming. It is the preferred control strategy when interference is scarce or unpredictable, and thus cannot be anticipated. Based on the dual mechanism of control theory, a differential impact of anxiety on reactive and proactive control can be predicted. Anxious people are expected to have less working memory capacity at their disposal because of the anxiety-related thoughts and physical stress and tension they experience, which occupy working memory resources. This makes it more difficult for anxious people to keep goal-relevant information active in working memory ^27,28^. As a result, their proactive control will be impaired and they will rely more on reactive control. Fales et al. ^29^ indeed showed that anxious people exhibited reduced sustained activity but increased transient activity in working memory brain regions (see also ^30^).

However, an alternative dynamic between anxiety and reactive versus proactive control also seems plausible. Reactive control is also more susceptible to interference from task-irrelevant internal or external sources ^27^. As this corroborates with the higher distractibility assumed in anxiety by the ACT, it could be that high anxious people experience difficulty during reactive control, where their distractibility is particularly cumbersome. Forster, Nunez Elizalde, Castle and Bishop ^31^ for example, not only observed a link between trait anxiety and impaired proactive control, but also between trait anxiety and impaired reactive control. People with a generalized anxiety disorder have also been reported to show a reduction in the general detection of interference in a flanker task where interference was unpredictable, and thus could not be proactively anticipated ^32^. If anxiety causes disruptions in interference detection, this should particularly hamper reactive control where control is only exerted after the interference is noticed (see also ^33^).

Suárez-Pellicioni, Núñez-Peña and Colomé ^34^ directly used the discrepancy between reactive and proactive control when investigating inhibition deficiencies in math anxious individuals. They studied event-related brain potentials (ERPs) for high and low math anxious individuals when performing a numerical Stroop task. In this task, number pairs differing in physical size had to be judged on numerical magnitude, creating interference when the physical size of the numbers conflicts with the numerical magnitude. Generally, people are slower and less accurate on interference compared to no interference trials (i.e., interference effect). High math anxious individuals showed a larger interference effect than low math anxious individuals, confirming their struggle with inhibiting irrelevant information (see also ^19,22^). With regards to the ERPs, clear effects were only obtained when also taking into account the previous Stroop trial. In general, people show a larger interference effect when no interference is present on the previous trial as opposed to when interference is present on the previous trial (i.e., conflict adaptation or the Gratton effect; ^35,36^). In the ERP analyses Suárez-Pellicioni et al. ^34^ found that this conflict adaptation effect was only present in the ERPs of high math anxious individuals. Based on this, the authors concluded that high math anxious individuals only reactively exerted control after incongruent trials, while low math anxious individuals relied more on a maintained, proactive control strategy.

However, the Stroop task used in the study of Suárez-Pellicioni et al. ^34^ was attuned to stimulating a reactive control strategy. Interference and no-interference trials were presented with equal proportions (i.e., 50%), creating a context where interference is completely unpredictable. This task context typically favours a reactive control strategy, as interference anticipation is non-optimal here (see for example ^37^ on how to distinguish reactive and proactive control using a Stroop task). Furthermore, it can be argued that conflict adaptation is an index of reactive rather than proactive control (see ^38^), making it a rather weak index of proactive control. Finally, as the ERP analyses were time-locked on the presentation of the Stroop stimulus, one could argue that the “proactive” control exerted by low math anxious individuals was simply a fast reactive control response to the stimulus and the “reactive” control exerted by high math anxious individuals a slow reactive control response to the stimulus. This then would actually indicate an impaired (i.e., delayed) reactive control in high math anxious individuals, rather than an increased reliance on reactive control.

In conclusion, math anxiety seems to be characterized by a deficient *general* inhibition, as predicted by the ACT. However, to establish precisely which inhibition processes (i.e., reactive or proactive) are affected by math anxiety, a task is needed that directly allows us to disentangle reactive and proactive control. The aim of the current study is precisely to offer such a new approach in this field. Specifically, in the current study we used an arrow flanker task ^39^ where adult participants categorize a central arrow as pointing to the left or to the right while inhibiting surrounding distractor arrows. Importantly, we manipulated the proportion of interference trials, creating a context that stimulated reactive control (i.e., where interference is scarce) and a context that stimulated proactive control (i.e., where interference is frequent). In line with the proposed theoretical frameworks and previous empirical results, we expected that math anxiety, measured by the Abbreviated Math Anxiety Scale ^40^, would be related to slower responses on interference trials, where inhibition is required. This should be reflected in an increasing interference effect with increasing math anxiety. Furthermore, if math anxiety is particularly detrimental for proactive control due to its high demands on working memory capacity, we expected that higher levels of math anxiety would be related to poorer performance (i.e., a larger interference effect) in the proactive context. However, if math anxiety is particularly detrimental for reactive control due to its distractibility, we expected that higher levels of math anxiety would be related to poorer performance (i.e., a larger interference effect) in the reactive context.

## Method

### Participants

Ninety-eight participants were recruited from the participant pool at the Vrije Universiteit Brussel. They received a course credit for participation. All participants provided written informed consent. One participant was excluded because of self-reported dyscalculia. Similar to previous flanker studies ^41,42^, three participants were excluded because they made more than 20% errors on the flanker task. Two participants were removed because their median RT was more than 2.5 *SD*’s above the group’s mean of median RTs. Thus, 92 participants were included for analysis (22 males, mean age = 19.0 ± 1.3 years). This study was approved by the Ethical Committee of the KU Leuven (G-2017 10 951) and was performed in accordance with the guidelines and regulations of the KU Leuven.

### Apparatus

For the flanker task, participants were seated in dimly lit private cubicles. Stimuli were presented on a 17-inch monitor (60 Hz, spatial resolution = 1280 × 1024) located approximately 75 cm from the subject. Stimulus presentation and response registration was controlled by E-Prime 2.0 ^43^.

### Material

*Inhibition.* An arrow flanker task was used consisting of a central target arrow flanked by two distractor arrows on both sides. Depending on the direction of central arrow and flankers, congruent (i.e., <  <  <  <  < and >  >  >  > >) and incongruent (i.e., <  <  >  <  < and >  >  <  > >) flanker stimuli (4° wide and 1° high) were created. They were presented in white against a black background in the centre of the screen. Participants were instructed to respond as fast and as accurately as possible in response to the central target arrow and to ignore the flankers. They had to press “a” on an Azerty keyboard with their left index finger when the central arrow was pointing to the left and “p” with their right index finger when the central arrow was pointing to the right. Each trial started with a 500 ms fixation cross, followed by a blank screen for 500 ms. This was followed by the flanker stimulus, which remained on the screen until the participant responded. No feedback was provided. Two blocks of 160 trials each were presented with different proportions of congruent and incongruent trials. In the mostly congruent (MC) block, the ratio of congruent:incongruent trials was 80:20; in the mostly incongruent (MI) block, the ratio of congruent:incongruent trials was 20:80 (see ^38^) . Participants are typically slower on incongruent compared to congruent trials (i.e., congruency effect), as the former contains interference that needs to be inhibited. The MC block creates a context where interference is scarce and unexpected, thereby triggering a reactive control strategy. The MI block creates a context where interference is frequent and can thus be anticipated, thereby triggering a proactive control strategy. This manipulation of proportion congruency typically leads to a decreased congruency effect in the MI compared to the MC block ^44^. This proportion congruency effect or PCE indicates that a proactive control mode is active on all trials (congruent and incongruent) in the MI block, which reduces the difference in RT between both trial types. In the MC block, reactive control is active only on incongruent trials. This is a late correction mechanism and therefore leads to slower responses on unexpected incongruent trials in the MC block, which leads to large congruency effects in the MC block compared to the MI block. This implies that the frequent interference in the MI block leads to enhanced cognitive control which decreases the congruency effect in this block compared to the MC block.

Presentation of the trials in each block was random. Participants were not informed about this manipulation of the proportion congruency. Each participant completed both block types. Block order (MC-MI or MI-MC) was counterbalanced across participants. Blocks were separated by a 30 s pause. Prior to the main experiment, participants completed 16 practice trials (50% congruent and 50% incongruent) with accuracy feedback.

*Math anxiety* was measured using the Dutch translation of the Abbreviated Math Anxiety Scale (AMAS; ^40^). The AMAS consists of nine items questioning how the respondent thinks (s)he would feel in a certain math-related situation (e.g., while taking a math exam). Responses need to be indicated on a 5-point Likert-type scale, ranging from 1 (low anxiety) to 5 (high anxiety). The total score represents the sum of the nine items (score ranging from 9 to 45, with higher scores indexing higher levels of math anxiety). Although to date, no data on the reliability and validity of the Dutch version of the AMAS have been reported, diverse foreign studies using translated versions of the AMAS have shown the validity, reliability and transcultural character of this questionnaire (e.g., ^16,45,46^). Previous studies have shown that the AMAS only moderately correlates with general anxiety (e.g., *r* = 0.35 in adults, ^47^; *r* = 0.37 to 0.41 for preadolescents, ^48^; *r* = 0.35 for adolescents, ^49^), suggesting a satisfactory divergent validity. For this sample, internal consistency measured using Cronbach’s *α* for the total score was 0.89.

*Mathematics achievement*. As math anxiety is confounded with math achievement ^7^, two pen-and-paper tests were included to control for the students’ mathematics achievement. As Ashcraft and Moore ^7^ have argued that math anxiety might have little effect on simple arithmetic, but larger effects on more complex math problems, we included both a simple and a more complex arithmetic test.

*Arithmetic fluency*. The arithmetic number fact test (Tempo Test Rekenen or TTR; ^50^) contains 200 simple arithmetic number fact problems presented in five columns (from left to right: addition, subtraction, division, multiplication, and mixed problems) of 40 items with an increasing difficulty level. Participants received 30 s per column, to solve as many problems as possible within a column. Their total score (out of 200) was the number of correct answers. The TTR is a standardized arithmetic test that is frequently used in the Flemish education system. For this sample, Cronbach’s *α* was 0.90.

*Arithmetic skills*. The Cognitive Developmental skills in aRithmetics, 5th grade (CDR-5, ^51^) assesses more complex arithmetic using nine different subskills. For the current study, four subtests were used to measure participants’ procedural calculation skills and abilities to solve applied word problems: the Procedural calculation subtest which consisted of five items where participants had to complete multi-step operations (e.g., 1263 + 861 + 73 + 445 = ?); the Mathematical reasoning subtest which consisted of five items where participants had to interpret and solve a procedural computation (e.g., 370.5 is 0.9 less than …); the Word problems with distraction which contained five items with additional item-irrelevant information (e.g., “Lisa has 2 marbles and 3 stickers. She gets 40 marbles. How many marbles does she have now?”); the Word problems without distraction which contained five items with only item-relevant information (e.g., “Emily has 40 marbles. She gives away 2 marbles. How many marbles does she have left?”). Participants were given 15 min time to complete all 20 items.. The number of correct answers was their total score (ranging from 0 to 20). For purposes unrelated to this study, participants also rated their own perceived performance on a scale from 0 to 20 at the end of the test. For this sample, Cronbach’s *α* was 0.75.

### Procedure

All participants first completed the inhibition task on a computer in individual cubicles. Afterwards, the pen-and-papers math tests and math anxiety questionnaire (i.e. the TTR, CDR and AMAS) were administered in groups of maximum 14 participants. The order of these pen-and-paper tasks was counterbalanced. Fifty participants first completed the TTR and CDR and filled in the AMAS afterwards. Forty-two participants filled in the AMAS first and then completed the TTR and CDR. This allowed us to control for the possible effects of completing math tasks first on reporting math anxiety afterwards and vice versa.

### Statistical analysis

Individual trial RTs (in ms) of the flanker task were analysed using a linear mixed model. The model included Congruency (congruent vs. incongruent) and Block (MC vs. MI) as fixed factors, and the standardized scores on math anxiety and on the indices of mathematics achievement (i.e., TTR and CDR) as covariates. The random effects structure was modelled stepwise (see Table 1). First, a model with only a random intercept for Subject was created (Model 1). Second, one model with an additional random slope for Congruency (Model 2) and one model with an additional random slope for Block (Model 3) were created. Third, a model with additional random slopes for both Congruency and Block was constructed (Model 4). Finally, a model with an additional interaction term for Congruency and Block was created (Model 5). Each augmented model was statistically compared to the previous one(s) using the likelihood ratio (χ2). Akaike information criterion (AIC; ^52^) is reported as a measure of model fit, with lower values indicating a better fit. The model with random effects structure that showed the best fit was selected. This turned out to be Model 5. Models were fitted using linear mixed models with the maximum likelihood procedure in the lme4 package ^53^ for R ^54^. Satterthwaite's adjustments were applied to obtain *t*-statistics with approximate degrees of freedom. Post-hoc comparisons were performed when appropriate by comparing the estimated marginal means (EMMs) for specified factors or factor combinations with Tukey corrections for multiple testing.

**Table 1:** Comparison of the models with different random effect structures.

	Model	Random factor	*df*	AIC	log lik	Test	χ^2^	*p*
RT	1	Subject*	18	338,683	−169,323			
	2	Subject*, Congruency°	20	337,556	−168,758	2 vs. 1	1130.25	< 0.001
	3	Subject*, Block°	20	338,015	−168,987	3 vs. 1	671.69	< 0.001
	4	Subject*, Congruency°, Block°	23	337,283	−168,619	4 vs. 2 4 vs. 3	279.15 737.71	< 0.001 < 0.001
	5	Subject*, Congruency°, Block°, Congruency × Block°	27	336,951	−168,449	5 vs. 4	339.71	< 0.001
Errors	1	Subject*	17	6437	−3201			
	2	Subject*, Congruency°	19	6416	−3189	2 vs. 1	24.62	< 0.001
	3	Subject*, Block°	19	6432	−3197	3 vs. 1	9.08	0.011
	4	Subject*, Congruency°, Block°	22	6412	−3184	4 vs. 2 4 vs. 3	10.14 25.68	0.017 < 0.001
	5	Subject*, Congruency°, Block°, Congruency × Block°	26	6410	−3179	5 vs. 4	10.47	0.033

Note. * = intercept; ° = slope.

Error rates were analysed similarly, using generalised linear mixed models with a logistic link function from the lme4 package ^53^. Again, model comparison revealed that Model 5 showed the best fit (Table 1). Since no adjustments of degrees of freedom are available for binary data, Wald’s *z* is reported instead of a *t*-value.

## Results

### Descriptive statistics

With regards to math anxiety, participants’ average score on the AMAS was 24.0 (*SD* = 6.8, range 11–42). The AMAS scores did not significantly differ between men and women (resp. 21.6 versus 24.7, *t*(87) = -1.85, *p* = 0.067). With regard to mathematics achievement, participants had average scores of 97.2 (*SD* = 16.5, range 36–149) and 10.4 (*SD* = 3.2, range 1–17) on the TTR and CDR, respectively. Table 2 shows the Pearson correlations between math anxiety, the two mathematics achievement measures, the overall mean median RTs on the inhibition task and mean error rates on the inhibition task. Performance on TTR and CDR were positively correlated. Interestingly, math anxiety did not significantly correlate with TTR or CDR performance. Performance on the CDR was negatively correlated with performance on the inhibition task, indicating that higher CDR scores were related to faster RTs and fewer errors on the inhibition task. Finally, slower responses on the flanker task correlated with lower error rates. The order of the tests (AMAS-TTR-CDR or TTR-CDR-AMAS) did not have an impact on math anxiety itself (*t*(87) = 1.39, *p* = 0.17), nor on the correlations between math anxiety and the two indices of mathematics achievement (i.e., partial correlations of *r* = -0.15, *p* = 0.17 between math anxiety and TTR and *r* = -0.14, *p* = 0.19 between math anxiety and CDR when controlling for the order of the test).

**Table 2:** Means (*SD*) and Pearson correlations between math anxiety, mathematics achievement measures (TTR and CDR), overall mean median reaction time (RTs) of correct responses on the inhibition task and overall mean error rate (ERR) on the inhibition task.

	Mean (*SD*)	Pearson correlations			
		TTR	CDR	RTs	ERR
Math Anxiety	24.0 (6.8)	−.14	−.16	.04	0.13
Mathematics achievement					
TTR	97.2 (16.5)		.52***	−.11	−0.13
CDR	10.4 (3.2)			−.24*	−0.24*
Inhibition task					
RTs	488.7 (46.3)				−0.30**
ERR	2.9 (2.5)				

*Note*. TTR = Tempo Test Rekenen; CDR = Cognitive Developmental skills in aRithmetics; *N* = 92 except for correlations with math anxiety where *N* = 89;*** *p* < .001; ** *p* < .01; * *p* < .05.

### RT analysis

The first trials of each block (1.3%), incorrect responses (2.9%), and responses exceeding 1500 ms (0.4%) were excluded from the RT analysis (for similar criteria see for example Aben et al., 2017). The constructed model, including the statistics, can be found in Table 3. This model showed a main effect of Congruency, indicating that subjects on average responded faster to congruent compared to incongruent trials (464 ms versus 608 ms. A main effect of Block was also found, with longer RTs on the MC than on the MI block (545 ms versus 528 ms). There was also a main effect of CDR score. One unit increase in CDR score, corresponded with a 21 ms decrease in RTs. An interaction between Congruency and Block was found. Larger congruency effects were found on the MC block (on average 211 ms) than on the MI block (on average 77 ms), indicating a proportion congruency effect (PCE). Block also interacted with CDR score. This effect entails a steeper decrease of RTs on the MC block (on average -28 ms) compared to the MI block (on average -13 ms), for every unit increase in CDR score. Congruency interacted with math anxiety. This effect entails a steeper increase of RTs on incongruent (on average 15 ms) compared to congruent trials (on average 0 ms) for every unit increase in math anxiety. In other words, the congruency effect increased with increasing math anxiety. Block also interacted with math anxiety. This effect entails a steeper increase of RTs on the MC block (12 ms) compared to the MI block (2 ms) for every unit increase in math anxiety.

**Table 3:** Fixed effects of the linear mixed model on RTs.

Predictor	Estimate	Std. Error	*df*	*t*	*p*
Intercept	536.96	6.25	84.98	85.95	< 0.001
Congruency	−72.24	3.15	84.96	−22.93	< 0.001
Block	8.83	2.30	84.70	3.84	< 0.001
TTR	1.16	7.24	84.94	0.16	0.87
CDR	−20.75	7.45	84.93	−2.78	0.007
Math anxiety	7.30	6.33	84.99	1.15	0.25
Congruency × Block	−33.72	2.17	84.58	−15.57	< 0.001
Congruency × TTR	2.03	3.65	84.80	0.56	0.58
Block × TTR	2.89	2.66	84.41	1.09	0.28
Congruency × CDR	6.86	3.76	84.76	1.83	0.071
Block × CDR	−7.72	2.74	84.33	−2.82	0.006
Congruency × math anxiety	−7.70	3.19	84.99	−2.41	0.018
Block × math anxiety	5.10	2.33	84.77	2.19	0.031
Congruency × Block × TTR	−1.45	2.51	84.25	−0.58	0.56
Congruency × Block × CDR	4.97	2.58	84.16	1.93	0.058
Congruency × Block × math anxiety	−5.62	2.20	84.65	−2.56	0.012

*Note*. TTR and CDR index mathematics achievement. TTR = Tempo Test Rekenen; CDR = Cognitive Developmental skills in aRithmetics.

**Figure 1:**
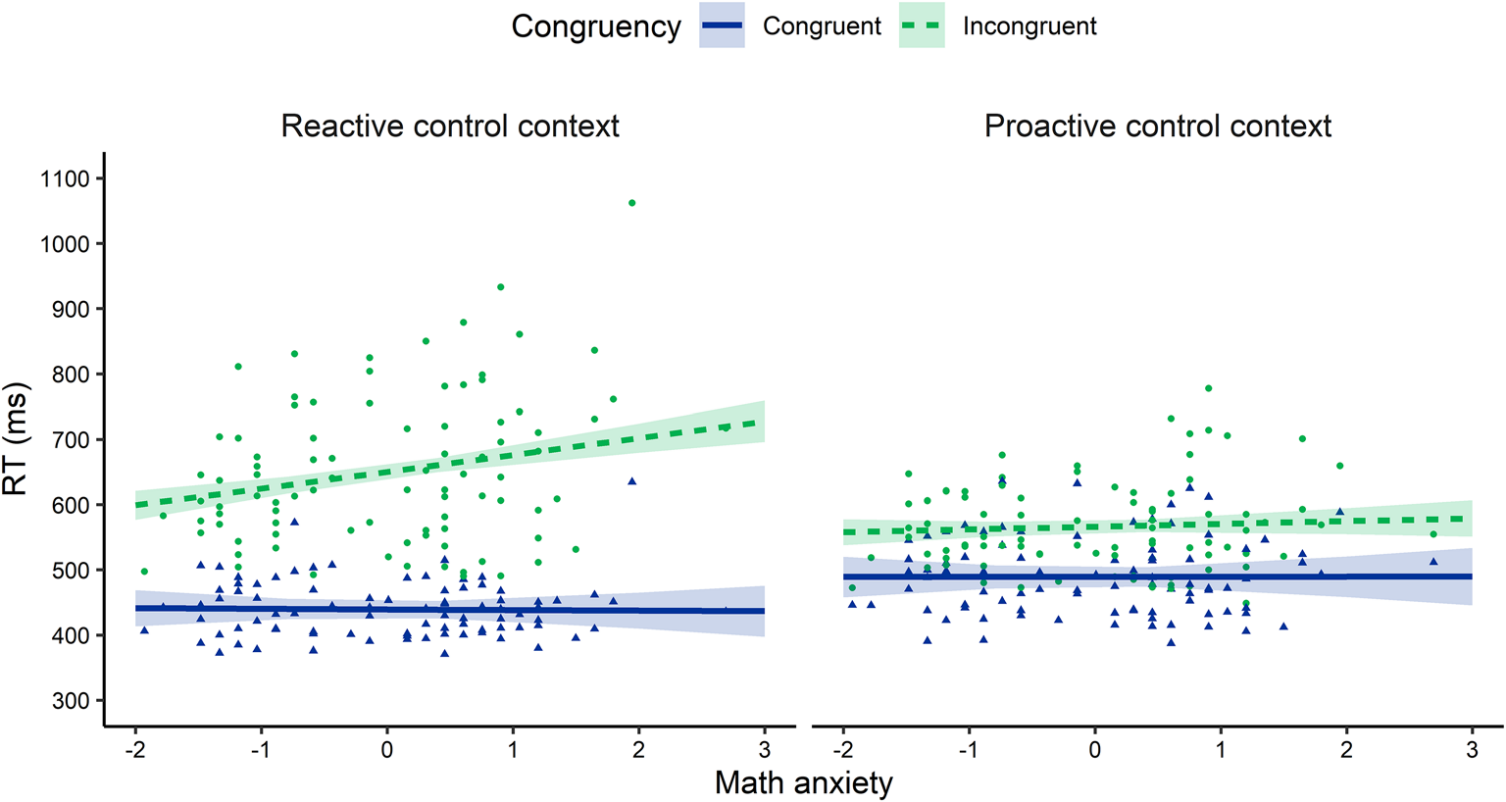
Visual presentation of the significant three-way interaction in the RT analysis. RTs are depicted per level of Block (the reactive control context or MC or the proactive control context or MI) and Congruency (Congruent or Incongruent) across (standardized) math anxiety. Dots represent mean RTs for each participant in each condition (thus, each participant is depicted four times on this graph, once for each Block × Congruency condition). Error bands represent 95% confidence limits.

Crucially, a three-way interaction was found between Congruency, Block, and math anxiety. This interaction is also depicted on Fig. 1. To break down this interaction, we computed estimated marginal means of the linear trend of the Congruency × math anxiety interaction. A significant Congruency × math anxiety interaction was found for the MC block, *t*(84.9) = 2.72*, p* = 0.008. The congruency effect in the MC block increased with 27 ms with every unit increase of math anxiety. No such interaction was found in the MI block, *t*(84.9) = 0.85, *p* = 0.40, indicating no relation between the congruency effect and math anxiety here. To provide evidence for the absence of this interaction between Congruency and math anxiety in the MI block, we computed Bayes Factors (BFs) using the lmBF package in R (https://cran.r-project.org/web/packages/BayesFactor/). First, a BF was computed for a model similar to model 5 fitted on only the MI data (BFMod1). Second, a BF was computed for the same model after excluding the Congruency × math anxiety interaction (BFMod2). Finally, the BF_10_ was computed according to BFMod1/BFMod2. This revealed BF_10_ = 0.1, indicating evidence in favor of the absence of an interaction between Congruency and math anxiety in the MI Block. The same analysis was repeated for the MC block. This revealed BF_10_ > 100, indicating strong evidence for the presence of an interaction between Congruency and math anxiety in the MC block. To further interpret this three-way interaction, the effect of math anxiety on RT was computed per condition (i.e., the estimated marginal means of linear trends of math anxiety in each of the four conditions, depicted in the slopes of the regression lines in Fig. 1). This revealed that this effect was significant only for the incongruent trials in the MC block (26 ms), *t*(84.9) = 2.27, *p* = 0.026. Indeed, tukey-corrected pairwise comparisons of the four slopes revealed that the effect of math anxiety on incongruent trials in the MC block was larger (26 ms) compared to the congruent trials in the MC block (-1 ms), *t*(84.9) = 2.72, *p* = 0.039, the congruent trials in the MI block (0 ms), *t*(84.8) = 2.82, *p* = 0.030, and the incongruent trials in the MI block (4 ms), *t*(84.7) = 2.68, *p* = 0.043. None of the other comparisons revealed significant differences (*p*s > 0.83).

### Error rate analysis

The first trial of each block (1.3%) and responses exceeding 1500 ms (0.4%) were excluded from the error rate analysis. The constructed model, including the statistics, can be found in Table 4. This model showed a main effect of Congruency: subjects committed more errors on incongruent (4.2%) than on congruent (0.4%) trials. A main effect of Block was also found: less errors were committed on the MC block (0.8%) than on the MI block (2.0%).

**Table 4:** Fixed effects of the linear mixed model on error rates.

Predictor	Estimate	Std. Error	*z*	*p*
intercept	−4.37	0.17	25.02	< 0.001
Congruency	−1.25	0.16	7.72	< 0.001
Block	0.47	0.16	−2.90	0.004
TTR	0.03	0.14	−0.22	0.82
CDR	−0.21	0.15	1.47	0.14
math anxiety	0.14	0.12	−1.19	0.23
Congruency × Block	−0.01	0.16	0.04	0.97
Congruency × TTR	0.05	0.11	−0.43	0.67
Block × TTR	−0.09	0.11	0.77	0.44
Congruency × CDR	−0.17	0.12	1.34	0.18
Block × CDR	0.13	0.12	−1.10	0.27
Congruency × math anxiety	0.07	0.10	−0.76	0.45
Block × math anxiety	0.02	0.09	−0.16	0.87
Congruency × Block × TTR	−0.11	0.11	1.01	0.31
Congruency × Block × CDR	0.09	0.12	−0.74	0.46
Congruency × Block × math anxiety	0.07	0.10	−0.75	0.45

*Note*. TTR and CDR index mathematics achievement. TTR = Tempo Test Rekenen; CDR = Cognitive Developmental skills in aRithmetics.

## Discussion

The aim of this study was to examine the effect of math anxiety on inhibition, a key cognitive control function associated with the central executive. Based on the dual mechanism of control theory ^27^ we were interested in disentangling the effect of math anxiety on two aspects of inhibition, namely proactive and reactive control. It has been proposed that as anxiety reduces working memory capacity, it should impair proactive control ^27,28^. However, the higher distractibility assumed in anxiety ^1^ could also lead to decreased performance in contexts where reactive control is needed, as exerting control in these contexts is particularly vulnerable to distraction.

Our results revealed that the congruency effect increased with increasing math anxiety. This confirms that inhibition (here: the suppression of interference caused by the irrelevant flankers on incongruent trials) is less efficient when math anxiety increases. This finding is in line with several previous studies (e.g., ^22,34^). Crucially, this impaired inhibition related to math anxiety was moderated by the type of inhibition that needed to be exerted. Specifically, in a reactive control context (i.e., an MC block), response times on incongruent trials increased with math anxiety. This indicates that the ability to inhibit interference caused by the flankers in the reactive context decreased with higher levels of math anxiety. Contrarily, in a proactive control context (i.e., an MI block), math anxiety did not influence response times on incongruent trials. Thus, inhibition of interference caused by the flankers in the proactive context was not influenced by math anxiety. Math anxiety was not related to response times on congruent trials in both blocks, which is to be expected as these trials do not contain interference that requires inhibition.

Consequently, although detriments in both proactive and reactive control have been suggested in anxiety, our results indicate impaired reactive control and intact proactive control when math anxiety increases. We are not the first to report a reduced reactive control in anxious people (e.g., ^31,32^). The factor that drives this mechanism might be distractibility. In the current reactive context (i.e., MC block), the flankers in the rare incongruent trials induce interference that requires inhibition that cannot be anticipated in advance and can only be elicited after the detection of the interference. In the intervals between interference detections, resources are freed up and can be allocated elsewhere, which is often efficient. However, this comes at a price: it makes reactive control vulnerable to attentional capture by other inputs (both internal and external), which could disrupt the ability to trigger inhibition when it is needed (i.e., on incongruent trials where interference is present; ^27^). Thus, the reactive task context in itself already increases the chances of distractibility. Additionally, anxiety is assumed to be characterized by high distractibility (e.g., ACT). According to the ACT, high anxious people will allocate their attentional resources broadly, decreasing their on-task focus and making them more susceptible to task-irrelevant distraction. We hypothesize that due to this state of high distractibility (triggered both by the context and by anxiety), the detection of interference, which is crucial for efficient reactive control, is reduced (e.g., ^32^), explaining the slower RTs we observed on incongruent trials in an MC block with increasing math anxiety.

We did not observe a relation between proactive control and math anxiety: proactive control remained intact with increasing math anxiety. The continuously maintained task goals in a proactive context protect against the influence of possible distractors ^27^. Thus, despite the higher distractibility in anxiety, this type of context might provide sufficient sustained focus on the task to avoid disturbance from internal or external sources of distraction. However, others have observed a primarily proactive deficiency in anxiety (e.g., ^26,29,34^). The dual mechanism of control theory predicts that anxiety would hamper the active maintenance of task goals in working memory, which should lead to impaired proactive control. It could therefore be that the demands that our current task made on working memory were not very high (as attested for example by the very low error rates and fast RTs) and therefore did not have to compete very strongly with the reduced working memory capacity assumed in anxiety. For example, Fales et al. ^29^ used a 3-back task, which is much more cognitively demanding than our arrow flanker task. Their fMRI results indicated that high-anxious participants reduced proactive control and increased reactive control using this task. Future research could manipulate working memory load or task difficulty to stimulate a more intense use of proactive control to ascertain whether proactive control is impaired in anxiety when placed under higher working memory constraints. Interestingly, we also did not observe main effects of math anxiety, indicating that general flanker task performance was not modulated by math anxiety. This is however in line with Fales et al. ^29^ who also did not observe general performance reductions in a high math anxious group. As the task context in our and Fales’ study was not math-related, one could argue that this might have impeded effects of math anxiety on general performance. Even when using a basic numerical context, general performance differences are not always observed between low and high math anxious individuals (see ^34^), indicating that to trigger general performance effects, a complex mathematical context is perhaps needed in addition to math-related cognitive processes.

As we used a math-unrelated flanker inhibition task, our results also provide further support for a domain-general rather than a math-specific inhibition deficiency in math anxiety, which is in line with several previous studies (e.g., ^19,22,23^). As we did not include an index of other anxiety constructs (e.g., general anxiety or test anxiety), our data do not permit us to conclude that the domain-general deficiency in reactive control that we observed is specific for math anxiety or can be generalized to anxiety more broadly. However, Mammarella et al. ^26^ did still observe a relation between math anxiety and impaired inhibition using math-unrelated material, while controlling for general anxiety. This provides some support for the suggestion that although there might be overlap with general anxiety, at least a part of math anxiety is specific. As suggested in the introduction, albeit speculative, this ‘specificity’ to math might refer to the math *content*, but perhaps also to *the cognitive processes involved in math tasks*. It might be the case that, through repeated experiences with math anxiety when performing math tasks, one becomes anxious or tensed towards these cognitive processes as well. As a consequence, when people have to conduct a task which heavily relies on similar cognitive processes – such as the flanker task in the current study – math anxiety is also triggered, despite the absence of a math-related content. Future studies should assess more directly whether math anxiety indeed can also transfer to more general cognitive processes involved in math.

Strikingly, no significant correlation was found between math anxiety and mathematics achievement in the current study. This is not in line with the math anxiety literature. A large-scale meta-analysis showed a significant relation between these two constructs in college students ^12^. For the TTR, the lack of a relationship is not very surprising, as previous studies have already indicated that math anxiety has little effect on very simple arithmetic, such as basic addition and multiplication performed by college students (e.g., ^8^). Note that in this study of Ashcraft and Faust ^8^, the problems were very comparable (or even more complex) as in the current TTR, and rely on skills that are extensively trained in primary school (e.g., tables of multiplication). However, math anxiety should influence more complex arithmetic problems which tax working memory more heavily, such as the problems included in the CDR ^7^. We can only speculate on why we did not observe a relation between the CDR and math anxiety. Perhaps the CDR, which is often used in samples with children (e.g., ^55^) or adults with learning disabilities (e.g., ^56^), does not impose a high enough load on working memory for student samples.

### Conclusion

The current study examined the effect of math anxiety on two aspects that can be identified in inhibition: reactive and proactive control. We showed that math anxiety specifically hampered reactive control. We hypothesized that this is due to a state of increased distractibility, evoked both by the reactive task context and by the presence of math anxiety. This state of distractibility could lead to an impaired detection of interference, which in turn causes slower response times on trials containing interference in a reactive context.

## Data Availability

The dataset generated and analysed during the current study is available in the OSF repository [https://osf.io/; 10.17605/OSF.IO/2FR7E].
